# Supplementary material for: Development and application of multiplex PCR for the rapid identification of four Fusarium spp. associated with Fusarium crown rot in wheat
Source: PeerJ. 2024 Jun 27;12:e17656. doi: 10.7717/peerj.17656 (PMC11214737; doi:10.7717/peerj.17656)
Supplement: Supplemental Information 1 [file peerj-12-17656-s001.docx]

**
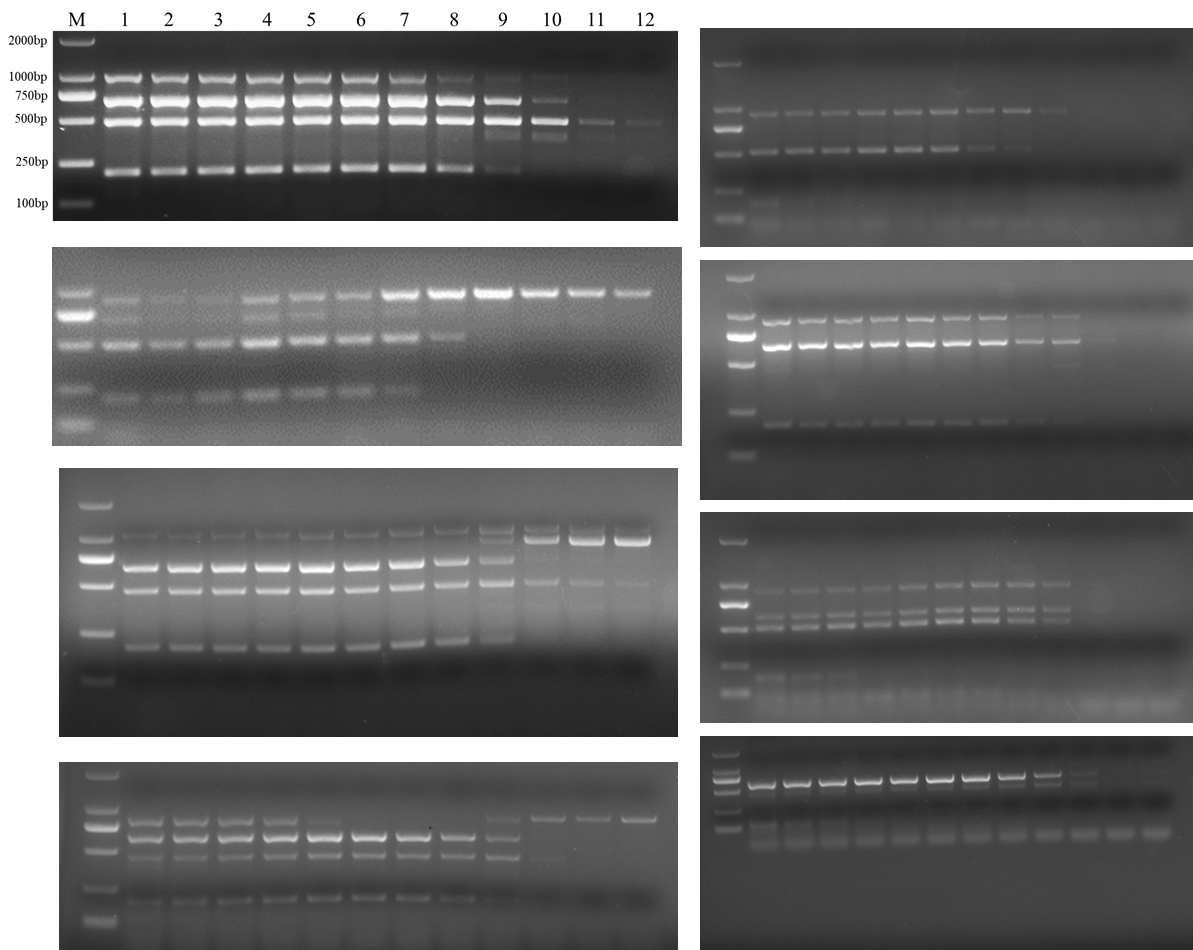
**

**Figure S1. Annealing temperature test of primer sets with different lengths and G+C content.** Gradients of annealing temperature. Lane M: 2000 bp DNA ladder, Lanes 1-12: 45, 46.1, 47.7, 50.5, 53, 55, 57.2, 59.4, 61.6, 63.4, 64.6 and 65 ℃.


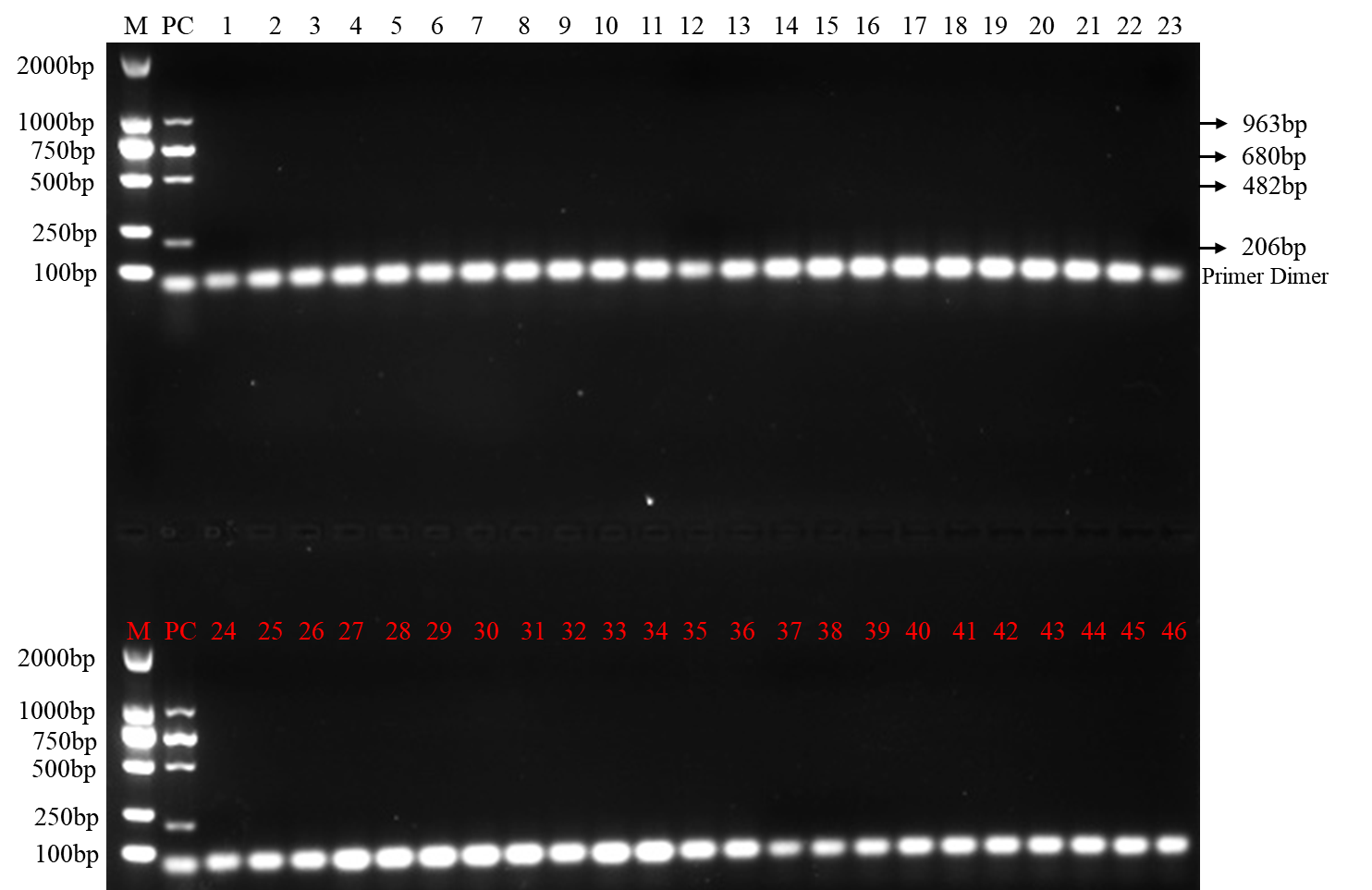


**Figure S2.** **Forty-six fungi pathogens with no amplified product.** M: DL2000 marker; PC: positive control; Lane 1-46: DNA of *Fusarium solani, Fusarium incarnatum, Fusarium equiseti, Fusarium oxysporum, Fusarium oxysporum, Fusarium oxysporum, Fusarium oxysporum, Fusarium oxysporum, Fusarium oxysporum, Fusarium humuli, Fusarium brachygibbosum, Fusarium fujikuroi, Alternaria alternata, Alternaria spp, Ascochyta pisi Libert, Botryophaeria dothidea, Botrytis cinerea, Botrytis cinerea, Cercospora kikuchii, Colletorichum lagenerium, Colletotrichum gloeosporioides, Diaporthe phaseolorum, Glomerella cingulata, Leptosphaeria biglobosa , Leptosphaeria maculans , Mycosphaerella melonis, Mycosphaerella melonis, Ophiostoma ulmi, Pestalotiopsis theae, Phellinidium lsulphurascens, Phialophora gregata, Phoma pinodella, Phoma spp, Phomopsis amygdali, Phomopsis fukushii, Phomopsis helianthi, Phomopsis longicolla, Phomopsis truncicola, Rhizoctonia cerealis, Rhizopus oryzae, Sclerotinia sclerotiorum, Sclerotium rolfsii, Stenocarpella maydis, Verticillium albo-atrum, Verticillium dahliae, Verticillium dahliae.*

**
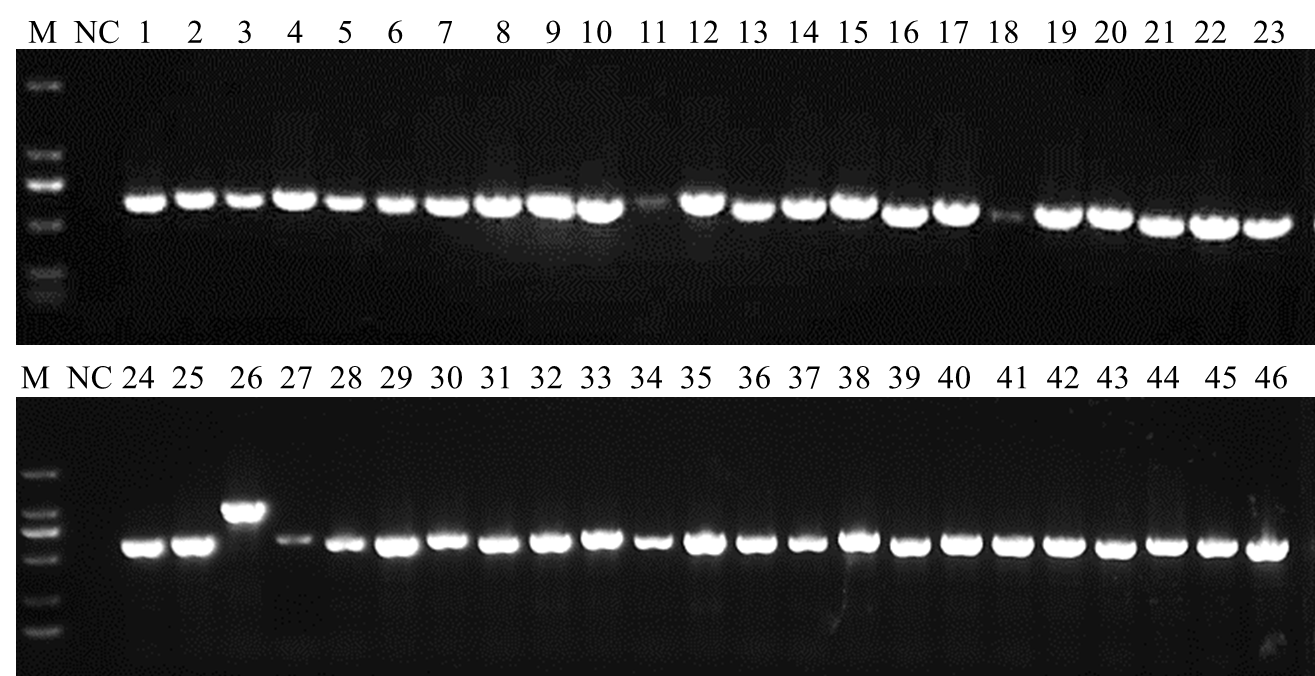
**

**Figure S3. Forty-six fungal DNA were amplified with ITS4/5 primers.** M: DL2000 marker; NC: negative control; Lane1-46: DNA of *Fusarium solani, Fusarium incarnatum, Fusarium equiseti, Fusarium oxysporum, Fusarium oxysporum, Fusarium oxysporum, Fusarium oxysporum, Fusarium oxysporum, Fusarium oxysporum, Fusarium humuli, Fusarium brachygibbosum, Fusarium fujikuroi, Alternaria alternata, Alternaria spp, Ascochyta pisi Libert, Botryophaeria dothidea, Botrytis cinerea, Botrytis cinerea, Cercospora kikuchii, Colletorichum lagenerium, Colletotrichum gloeosporioides, Diaporthe phaseolorum, Glomerella cingulata, Leptosphaeria biglobosa , Leptosphaeria maculans , Sclerotinia sclerotiorum, Mycosphaerella melonis, Mycosphaerella melonis, Ophiostoma ulmi, Pestalotiopsis theae, Phellinidium lsulphurascens, Phialophora gregata, Phoma pinodella, Phoma spp, Phomopsis amygdali, Phomopsis fukushii, Phomopsis helianthi, Phomopsis longicolla, Phomopsis truncicola, Rhizoctonia cerealis, Rhizopus oryzae, Sclerotium rolfsii, Stenocarpella maydis, Verticillium albo-atrum, Verticillium dahliae, Verticillium dahliae.*


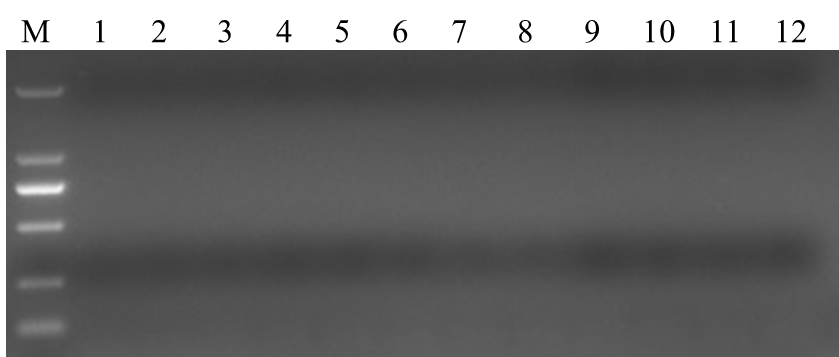


**Figure S4. No bands were amplified by the primer set in PCR system without DNA template.** M: DL2000 marker; Lane 1-12: no template controls.

**Table S1.** Final concentration of primers in the multiplex PCR.

|  | **Fu-4F** | | **Fgram-R** | | **Fpseu-R** | | **Fprol-R** | | **Fvert-R** | |
| --- | --- | --- | --- | --- | --- | --- | --- | --- | --- | --- |
|  | Final Conc. | Dosage | Final Conc. | Dosage | Final Conc. | Dosage | Final Conc. | Dosage | Final Conc. | Dosage |
|  | umol/L | ul | umol/L | ul | umol/L | ul | umol/L | ul | umol/L | ul |
| **Group Ⅰ** | 0.05 | 0.25 | 0.05 | 0.25 | 0.05 | 0.25 | 0.05 | 0.25 | 0.05 | 0.25 |
|  | 0.1 | 0.5 | 0.1 | 0.5 | 0.1 | 0.5 | 0.1 | 0.5 | 0.1 | 0.5 |
|  | 0.15 | 0.75 | 0.15 | 0.75 | 0.15 | 0.75 | 0.15 | 0.75 | 0.15 | 0.75 |
|  | 0.2 | 1 | 0.2 | 1 | 0.2 | 1 | 0.2 | 1 | 0.2 | 1 |
| **Group Ⅱ** | 0.1 | 0.5 | 0.05 | 0.25 | 0.05 | 0.25 | 0.05 | 0.25 | 0.05 | 0.25 |
|  | 0.2 | 1 | 0.1 | 0.5 | 0.1 | 0.5 | 0.1 | 0.5 | 0.1 | 0.5 |
|  | 0.3 | 1.5 | 0.15 | 0.75 | 0.15 | 0.75 | 0.15 | 0.75 | 0.15 | 0.75 |
|  | 0.4 | 2 | 0.2 | 1 | 0.2 | 1 | 0.2 | 1 | 0.2 | 1 |
| **Group Ⅲ** | 0.15 | 0.75 | 0.05 | 0.25 | 0.05 | 0.25 | 0.05 | 0.25 | 0.05 | 0.25 |
|  | 0.3 | 1.5 | 0.1 | 0.5 | 0.1 | 0.5 | 0.1 | 0.5 | 0.1 | 0.5 |
|  | 0.45 | 2.25 | 0.15 | 0.75 | 0.15 | 0.75 | 0.15 | 0.75 | 0.15 | 0.75 |
|  | 0.6 | 3 | 0.2 | 1 | 0.2 | 1 | 0.2 | 1 | 0.2 | 1 |
| **Group Ⅳ** | 0.2 | 1 | 0.05 | 0.25 | 0.05 | 0.25 | 0.05 | 0.25 | 0.05 | 0.25 |
|  | 0.4 | 2 | 0.1 | 0.5 | 0.1 | 0.5 | 0.1 | 0.5 | 0.1 | 0.5 |
|  | 0.6 | 3 | 0.15 | 0.75 | 0.15 | 0.75 | 0.15 | 0.75 | 0.15 | 0.75 |
|  | 0.8 | 4 | 0.2 | 1 | 0.2 | 1 | 0.2 | 1 | 0.2 | 1 |

The initial concentration of each primer is 10umol/L. The total reaction volume is 50ul.

**Table S2.** Multiplex PCR detection of target pathogen DNA within wheat samples.

| **Location** | **Host** | **Sample** | **Results of the multiplex PCR detection^a^** | | | |
| --- | --- | --- | --- | --- | --- | --- |
|  |  |  | **Fgram^b^** | **Fpseu^b^** | **Fprol^b^** | **Fvert^b^** |
|  | PC |  | + | + | + | + |
|  | NC |  | − | − | − | − |
| **Field wheat samples** | | | | | | |
| Xiangyang,  Hubei Province | Wheat | 1 | + | + | − | − |
|  | Wheat | 2 | + | + | − | − |
|  | Wheat | 3 | + | + | − | − |
|  | Wheat | 4 | + | + | − | − |
|  | Wheat | 5 | − | + | − | − |
|  | Wheat | 6 | − | − | − | − |
|  | Wheat | 7 | − | − | − | − |
|  | Wheat | 8 | − | − | − | − |
|  | Wheat | 9 | − | + | − | − |
| Suizhou, Hubei Province | Wheat | 10 | + | + | − | + |
|  | Wheat | 11 | − | + | − | − |
|  | Wheat | 12 | − | + | − | + |
|  | Wheat | 13 | − | + | − | + |
|  | Wheat | 14 | + | + | − | − |
|  | Wheat | 15 | + | + | − | − |
|  | Wheat | 16 | + | + | − | − |
|  | Wheat | 17 | + | + | − | − |
|  | Wheat | 18 | + | + | − | − |
|  | Wheat | 19 | − | − | − | − |
|  | Wheat | 20 | − | − | − | − |
|  | Wheat | 21 | − | − | − | − |
|  | Wheat | 22 | − | − | − | − |
| **Artificially inoculate wheat samples** | | | | | | |
|  | Wheat | 1 | + | + | + | + |
|  | Wheat | 2 | − | + | − | − |
|  | Wheat | 3 | − | + | − | − |
|  | Wheat | 4 | + | − | + | + |
|  | Wheat | 5 | − | − | + | + |
|  | Wheat | 6 | − | + | − | − |
|  | Wheat | 7 | − | + | − | − |
|  | Wheat | 8 | − | + | − | − |
|  | Wheat | 9 | + | − | − | − |
|  | Wheat | 10 | − | − | − | − |
|  | Wheat | 11 | − | − | − | − |
|  | Wheat | 12 | + | − | − | − |
|  | Wheat | 13 | + | − | − | − |
|  | Wheat | 14 | − | − | − | − |
|  | Wheat | 15 | − | − | − | − |
|  | Wheat | 16 | − | − | − | − |
|  | Wheat | 17 | + | + | − | − |
|  | Wheat | 18 | + | + | − | − |
|  | Wheat | 19 | + | + | + | + |
|  | Wheat | 20 | − | − | − | − |
|  | Wheat | 21 | − | − | − | − |
|  | Wheat | 22 | − | + | − | − |
|  | Wheat | 23 | − | + | − | − |
|  | Wheat | 24 | − | + | − | − |

^a^ Results of the multiplex PCR detection are indicated as positive (+) and negative (−).

^b^ Fgram: *Fusarium graminearum*; Fpseu: *Fusarium pseudograminearum*; Fprol: *Fusarium proliferatum*; Fvert: *Fusarium verticillioides*.
