# Supplementary material for: Development and application of multiplex PCR for the rapid identification of four Fusarium spp. associated with Fusarium crown rot in wheat
Source: PeerJ. 2024 Jun 27;12:e17656. doi: 10.7717/peerj.17656 (PMC11214737; doi:10.7717/peerj.17656)
Supplement: Supplemental Information 2 — Original electrophoretic images of all multiplex PCR assays. [file peerj-12-17656-s002.pdf]

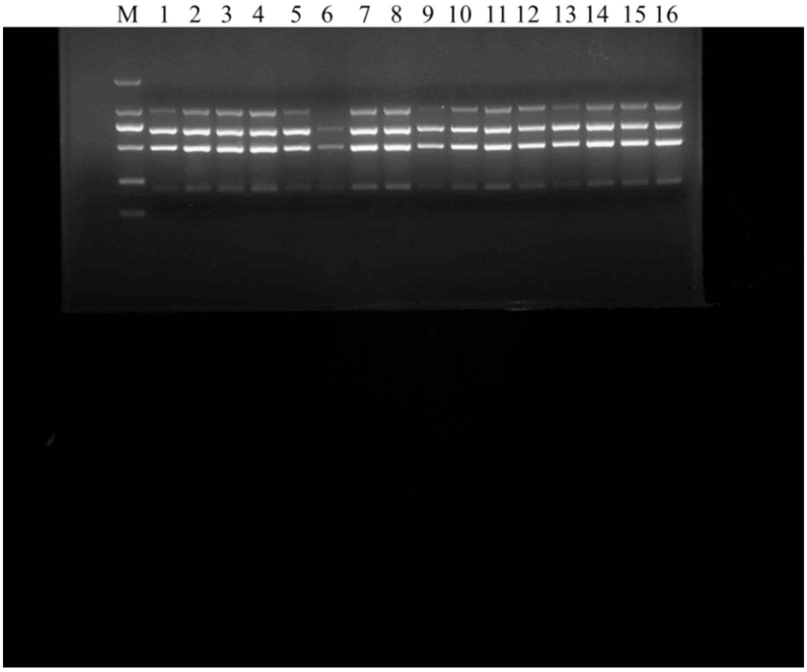

Figure 2A

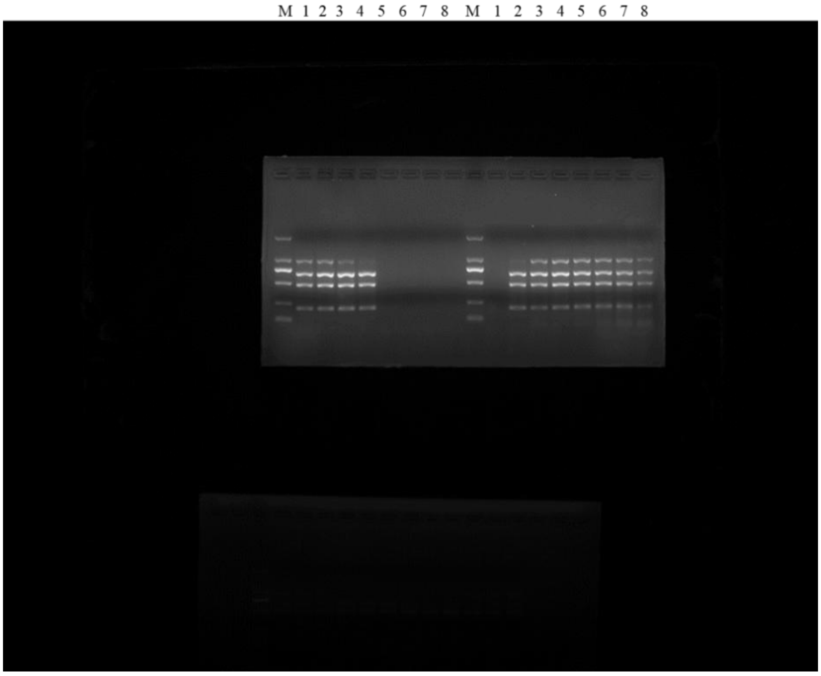

Figure 2B. Right and Figure 2C. Left.

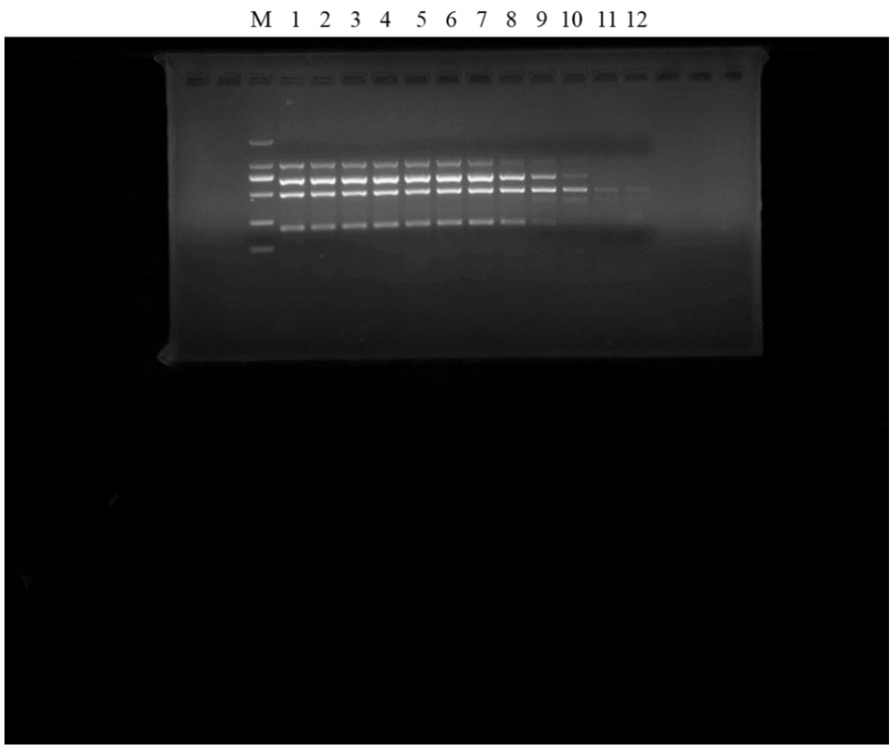

Figure 2D

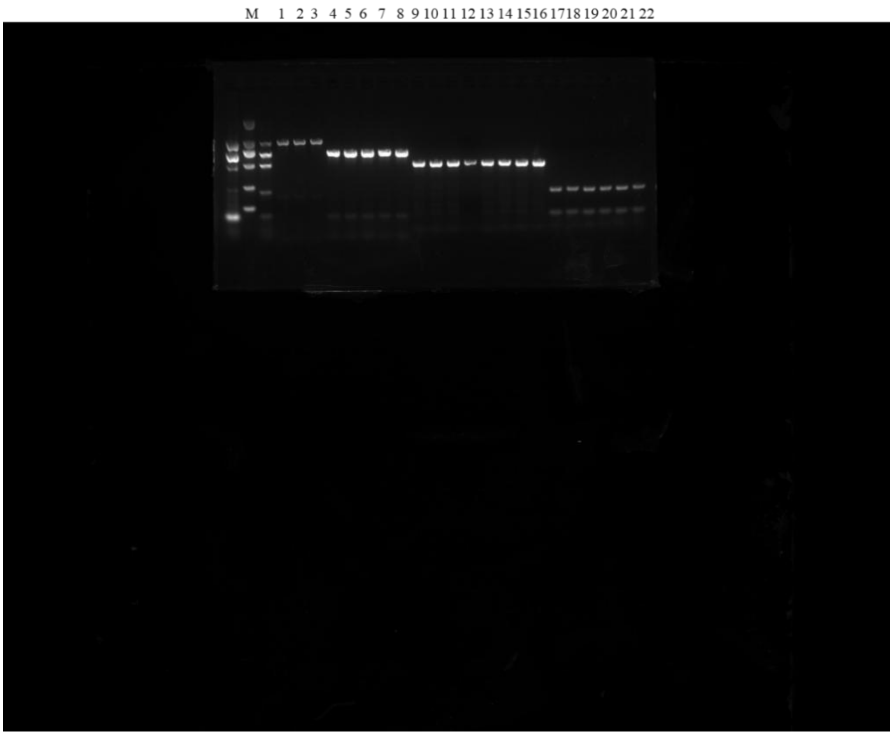

Figure 3

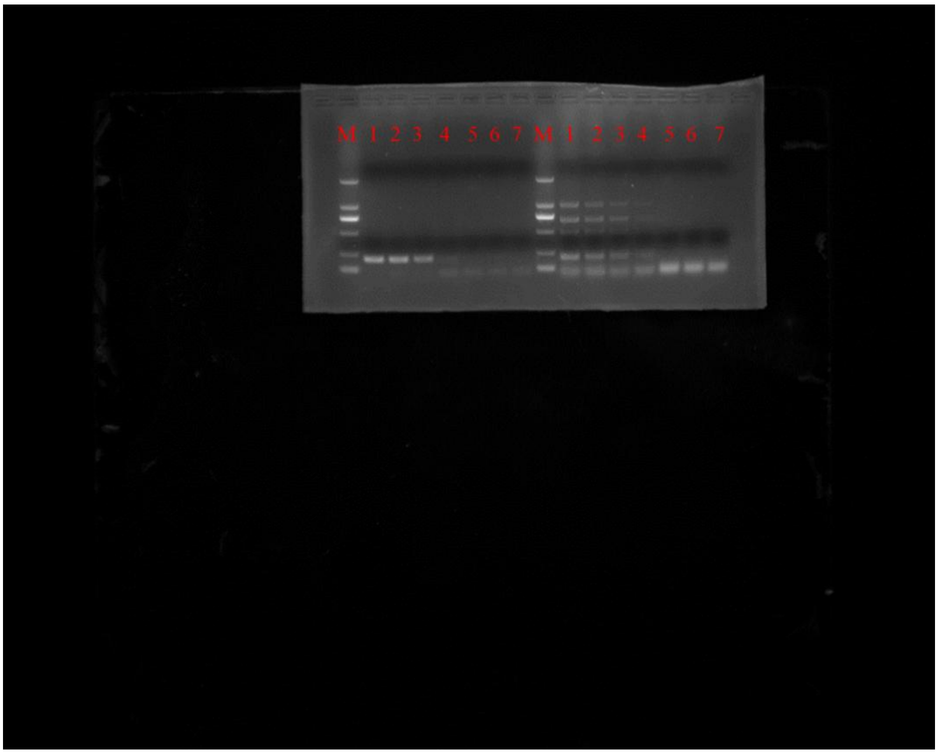

Figure 4A Right and Figure 4E Left

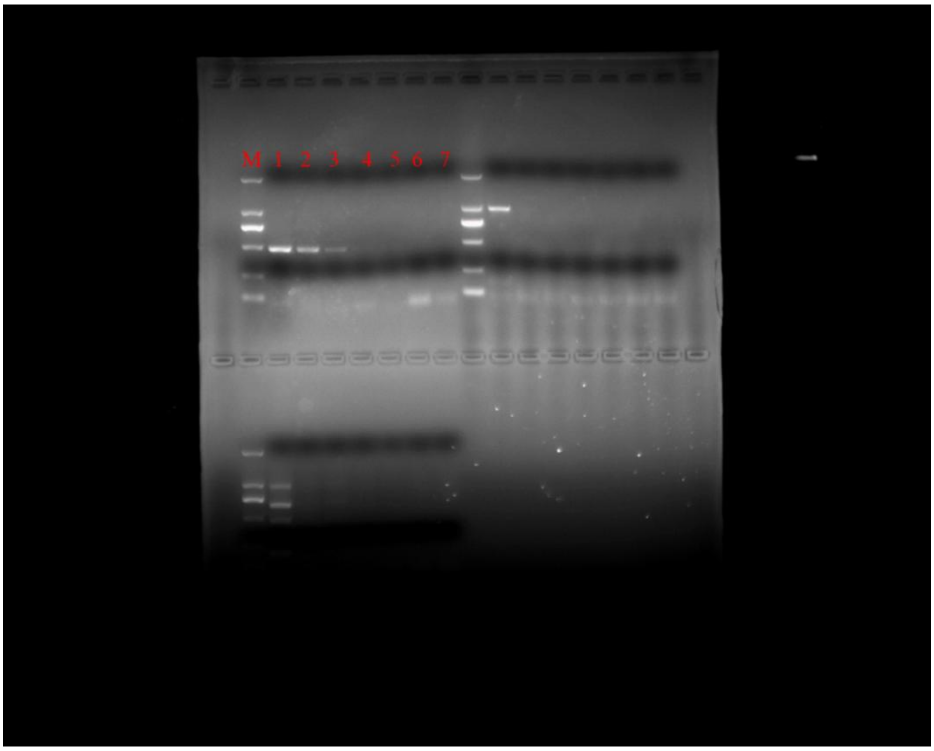

Figure 4D

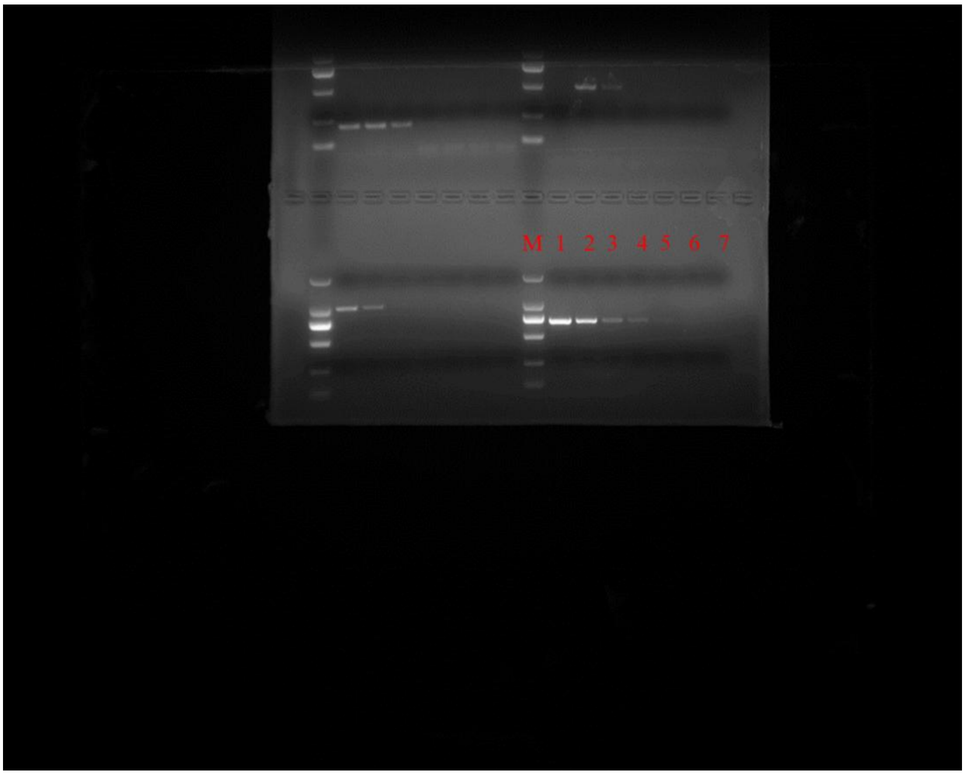

Figure 4C

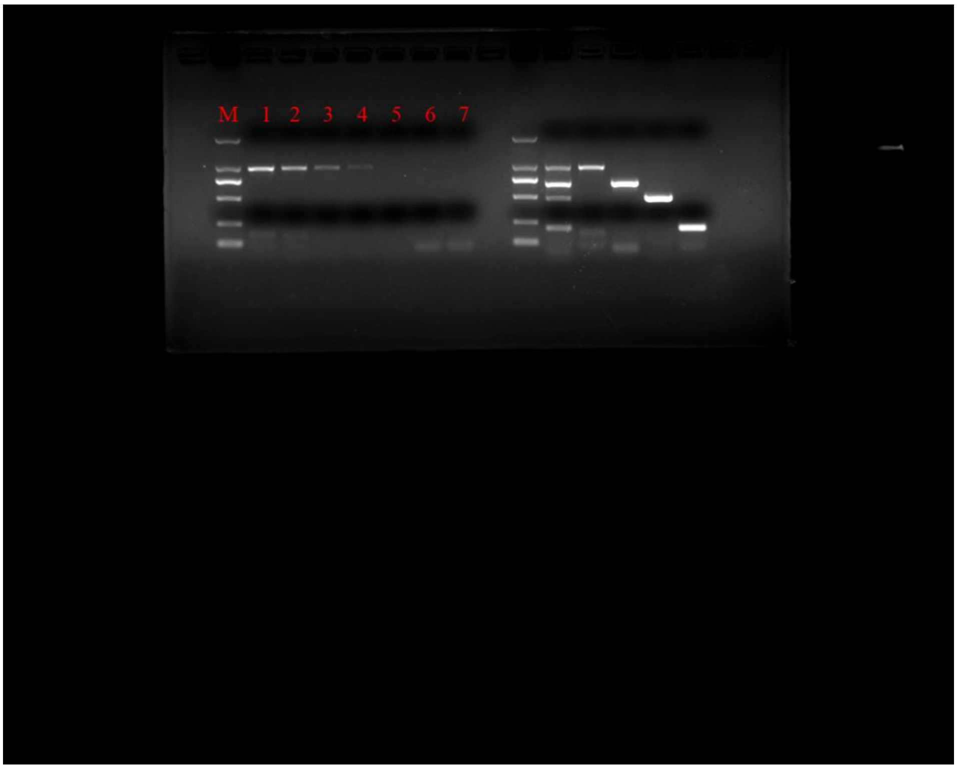

Figure 4B

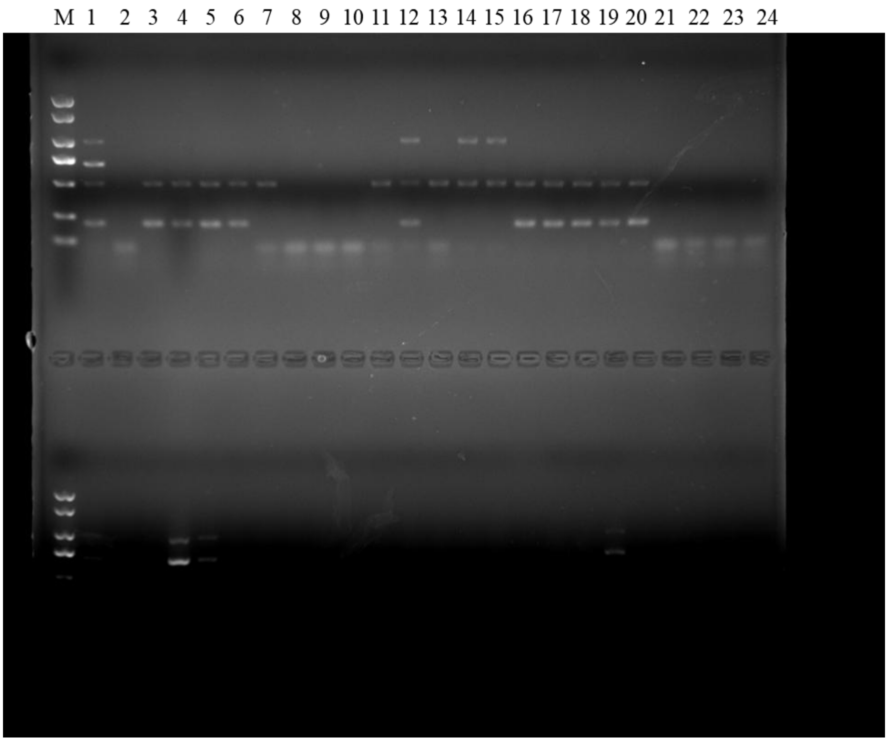

Figure 5A

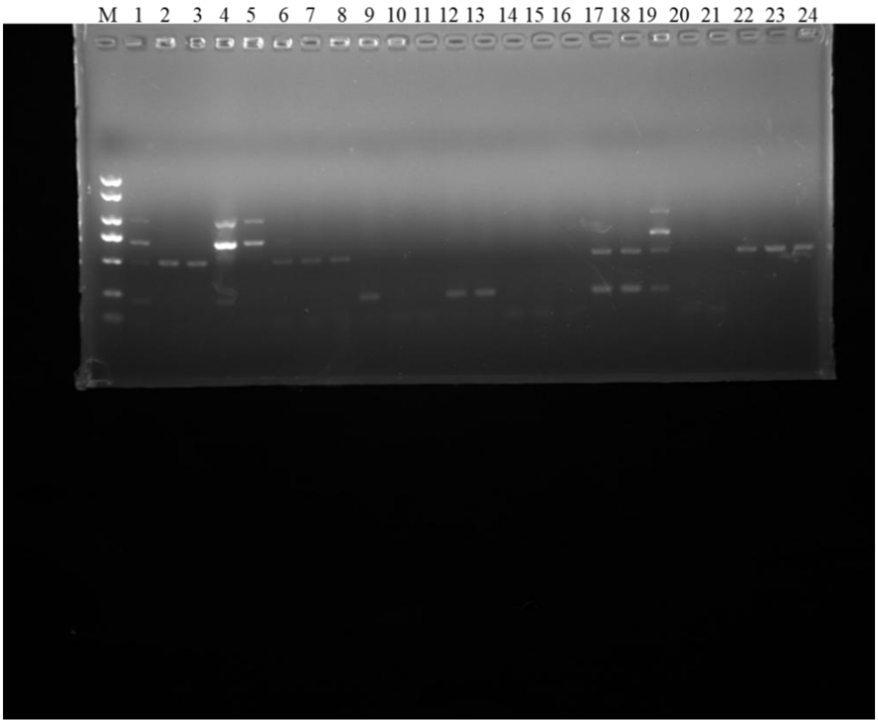

Figure 5B

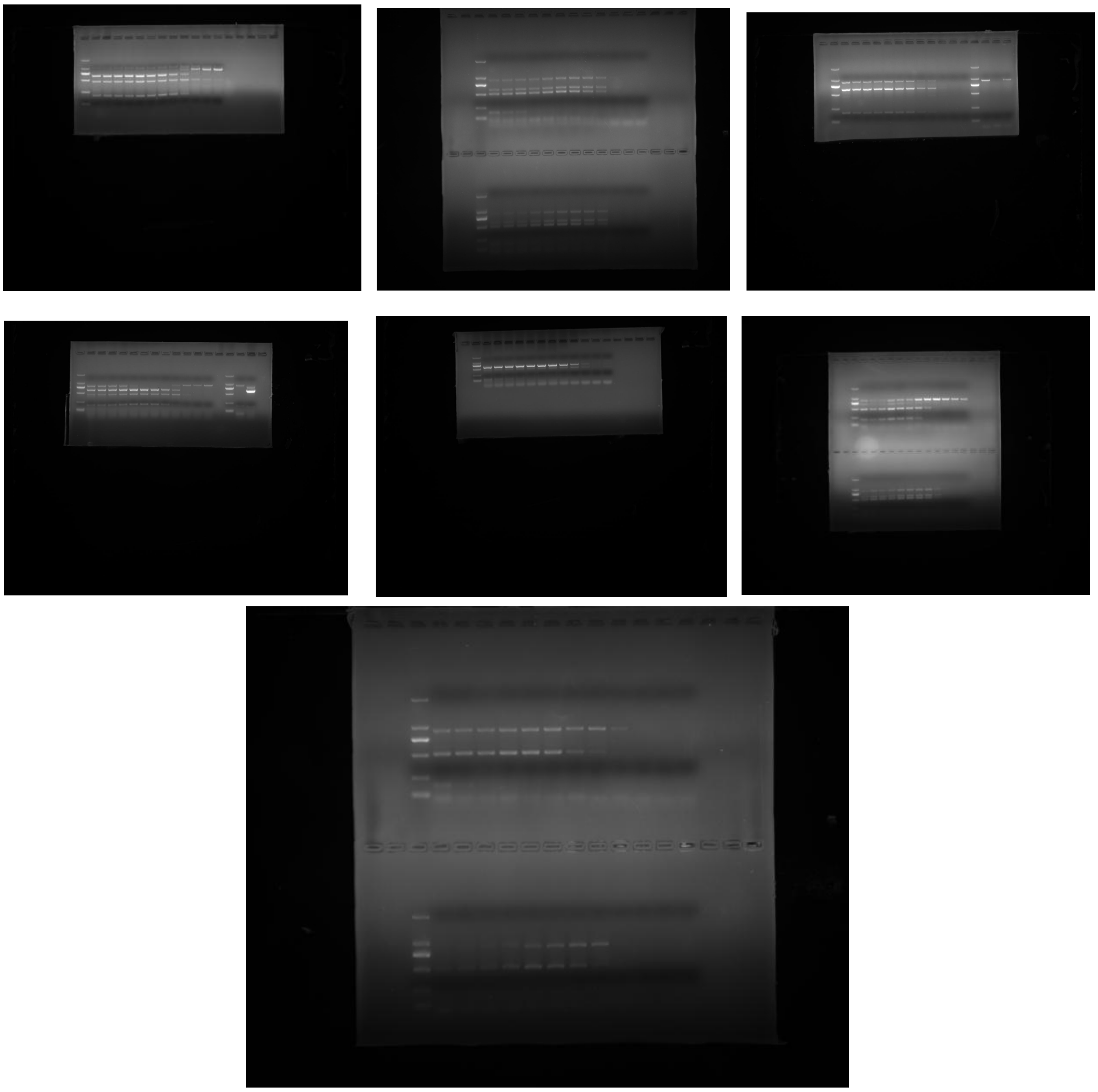

Figure S1

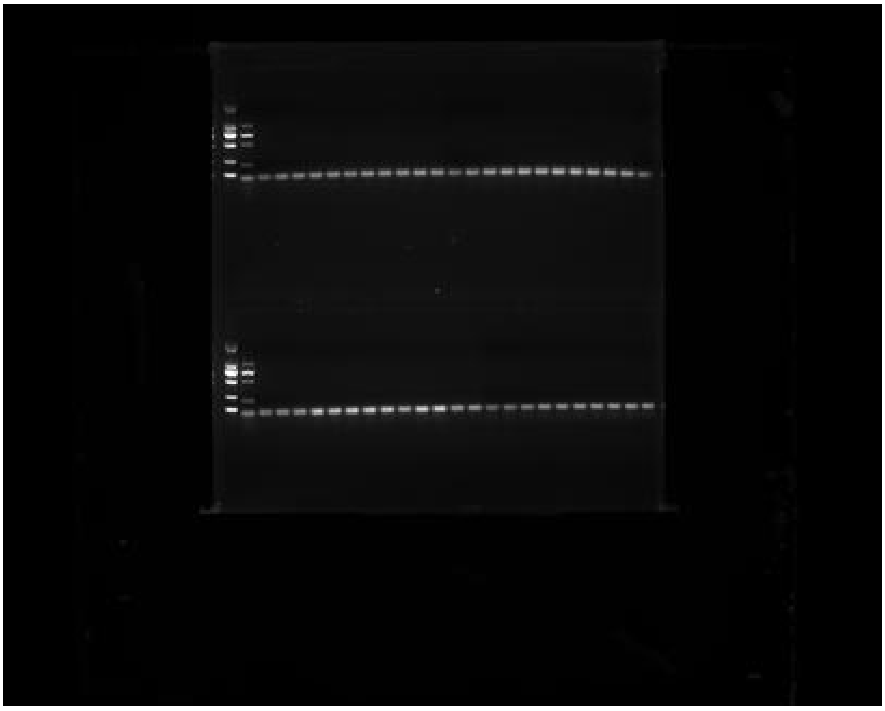

Figure S2

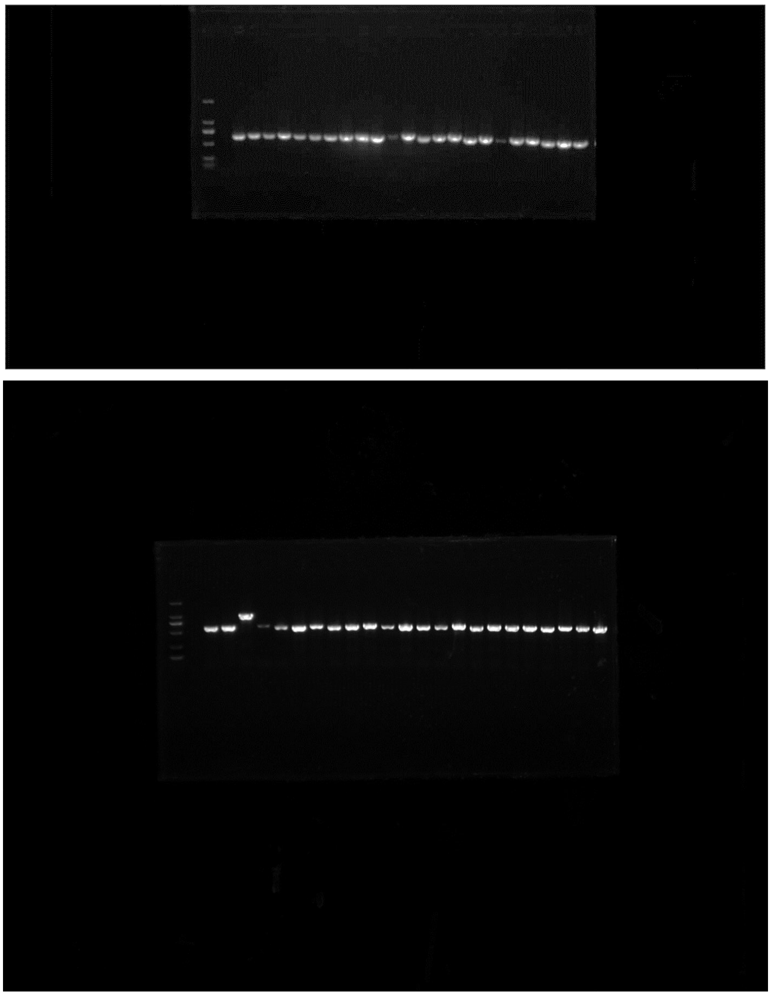

Figure S3

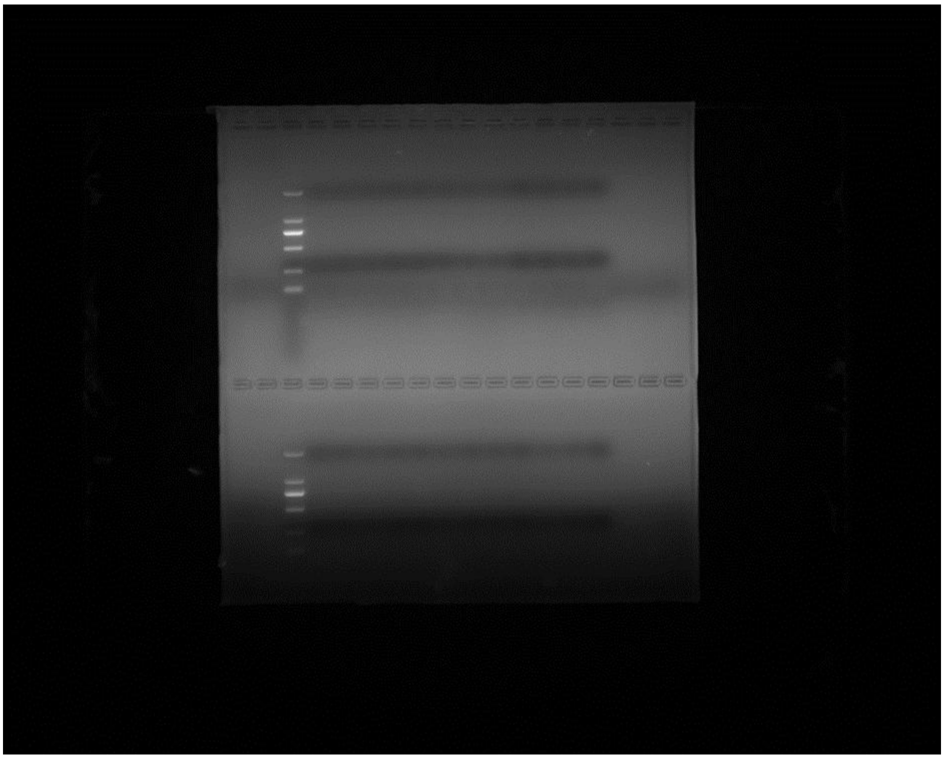

Figure S4
